# Molecular Diversity Analysis and Genetic Mapping of Pod Shatter Resistance Loci in *Brassica carinata* L.

**DOI:** 10.3389/fpls.2017.01765

**Published:** 2017-11-30

**Authors:** Rosy Raman, Yu Qiu, Neil Coombes, Jie Song, Andrzej Kilian, Harsh Raman

**Affiliations:** ^1^Graham Centre for Agricultural Innovation (an alliance between NSW Department of Primary Industries and Charles Sturt University), Wagga Wagga Agricultural Institute, Wagga Wagga, NSW, Australia; ^2^Wagga Wagga Agricultural Institute, NSW Department of Primary Industries, Wagga Wagga, NSW, Australia; ^3^Diversity Arrays Technology Pty. Ltd., University of Canberra, Canberra, ACT, Australia

**Keywords:** pod shattering, resistance, genetic mapping, Ethiopian mustard, QTL, molecular markers

## Abstract

Seed lost due to easy pod dehiscence at maturity (pod shatter) is a major problem in several members of Brassicaceae family. We investigated the level of pod shatter resistance in Ethiopian mustard (*Brassica carinata*) and identified quantitative trait loci (QTL) for targeted introgression of this trait in Ethiopian mustard and its close relatives of the genus *Brassica*. A set of 83 accessions of *B. carinata*, collected from the Australian Grains Genebank, was evaluated for pod shatter resistance based on pod rupture energy (RE). In comparison to *B. napus* (RE = 2.16 mJ), *B. carinata* accessions had higher RE values (2.53 to 20.82 mJ). A genetic linkage map of an F_2_ population from two contrasting *B. carinata* selections, BC73526 (shatter resistant with high RE) and BC73524 (shatter prone with low RE) comprising 300 individuals, was constructed using a set of 6,464 high quality DArTseq markers and subsequently used for QTL analysis. Genetic analysis of the F_2_ and F_2:3_ derived lines revealed five statistically significant QTL (LOD ≥ 3) that are linked with pod shatter resistance on chromosomes B1, B3, B8, and C5. Herein, we report for the first time, identification of genetic loci associated with pod shatter resistance in *B. carinata*. These characterized accessions would be useful in *Brassica* breeding programs for introgression of pod shatter resistance alleles in to elite breeding lines. Molecular markers would assist marker-assisted selection for tracing the introgression of resistant alleles. Our results suggest that the value of the germplasm collections can be harnessed through genetic and genomics tools.

## Introduction

The Ethiopian mustard [syn. Abyssinian mustard; *Brassica carinata* *A. Braun*. (2n = 4× = 34); genome B_c_B_c_C_c_C_c_], is an important leafy vegetable and oilseed crop in northeast Africa (Warwick et al., 2006). It is evolved as a result of a few interspecific hybridization events between *Brassica nigra* (BB genome, 2n = 2 × = 16) and *Brassica oleracea* (CC genome, 2n = 2× = 18) in Ethiopia. In recent years, this crop is also being utilized for biodiesel production due to its fatty acid composition. In addition, *B. carinata* harbors several genes for resistance to lodging, diseases, and pod shattering; and tolerance to abiotic stresses (Getinet et al., 1996; Taylor et al., 2002; Malik, 2008; Enjalbert et al., 2013; Wei et al., 2016; Sharma et al., 2017), which make it also an ideal candidate for broadening the narrow genetic base of canola – the world’s second largest oilseed crop (Cowling, 2007).

Dehiscence of fruiting structures is an orchestrated natural mechanism for seed dispersal and survival of many plant species. In Ethiopian mustard and other members of the Brassicaceae family, a dehiscence zone (DZ) is developed between the two valves and the replum, as the pods mature. The highly differentiated cells in DZ weaken the strength of the pods, leading to seed dispersal at maturity. Pod shattering is a highly undesirable trait for commercial seed production in *Brassica* crops and causes significant yield losses of up to 70% in canola (Colton and Potter, 1999). Generally, oilseed Brassicas are ‘windrowed’ to reduce seed loss due to shattering but this practice is not completely effective (Mongkolporn et al., 2003). Seed losses accelerate further with the prevalence of high wind velocity and extremely high temperatures during the time of harvesting in Australia. One of the foci of many *Brassica* breeding programs is to develop improved varieties for resistance to pod shattering so the standing crop can be directly harvested with combines without any significant seed loss.

Natural variation for shatter resistance exists in the *B. rapa, B. juncea, B. napus*, and *B. carinata* germplasm (Kadkol et al., 1985, 1986b; Prakash and Chopra, 1988; Mongkolporn et al., 2003; Wang et al., 2007; Raman H. et al., 2014; Zhang et al., 2016). However, shatter resistance in *B. napus* germplasm is insufficient to reduce yield loss under severe weather conditions (Raman H. et al., 2014). *B. carinata* is reported to be more resistant to seed shattering than *B. napus, B. rapa*, and, *B. juncea* (Zhang et al., 2016). Interestingly, *B. carinata* is also known to hybridize with the A*_r_* (*B. rapa*) and A_n_C_n_ (*B. napus*) genome species and produce viable ‘new type’ napus plants (A_n_A_n_C_n/c_C_n/c_) with diverse C_c_ genome (Navabi et al., 2010; Tian et al., 2010; Banga et al., 2011; Dhaliwal et al., 2017). This knowledge prompted us to characterize genetic variation and identify genetic loci for pod shatter resistance in *B. carinata* to improve the level of shattering resistance in other *Brassica* crops, especially in canola.

The testing of germplasm for pod shatter resistance under field conditions is often practiced in breeding programs but it is unreliable and confounded with growing environment. However, the availability of test methods like the random impact test and pendulum test to assess the pod strength have made possible the assessment of germplasm to categorize them into shatter tolerant/susceptible under laboratory conditions (Kadkol et al., 1985; Liu et al., 1994; Hossain et al., 2011). The pendulum test relies on the inherent difference in pod strength measured as ‘energy used to rupture pods’ [rupture energy (RE)] (Liu et al., 1994).

In *B. rapa* and *B. napus*, loci for pod shatter resistance have been delineated using molecular markers (Mongkolporn et al., 2003; Hu et al., 2012; Wen et al., 2013; Raman H. et al., 2014; Liu et al., 2016). For example, Raman H. et al. (2014) reported that several quantitative trait loci (QTL) on chromosomes A03, A09, A10, and C03 account for genetic variation in shatter resistance in the doubled haploid (DH) population derived from BLN2762/Surpass400 as well as in a diverse panel of 181 lines of *B. napus*. Subsequently, Liu et al. (2016) identified six significant QTL for resistance to pod shatter located on chromosomes A01, A06, A07, A09, C02, and C05 in a diverse panel of 143 *B. napus* accessions, and bi-parental DH and intermated populations derived from the maternal parent, ‘R1’ (resistant to pod shattering) and the paternal parent, ‘R2’ (prone to pod shattering). Both these described studies showed that at least one consistent locus on linkage group A09, which maps in the vicinity of *AUXIN RESPONSIVE REGULATOR 18* (*ARR18*) and MADS-box gene, *SHATTERPROOF (BnShp1)*, controls pod shatter resistance in Australian and Chinese germplasm. Several genes which are involved in a complex regulatory network, such as *SHATTERPROOF1 (SHP1)*; *SHATTERPROOF2 (SHP2)*; *FRUITFULL (FUL)*; *INDEHISCENT (IND)*; *ALCALTRAZ (ALC)*; and *REPLUMLESS (RPL)*, control pod shatter resistance in *Arabidopsis thaliana*, and other heterologous systems (Ferrandiz et al., 2000; Rajani and Sundaresan, 2001; Roeder et al., 2003; Liljegren et al., 2004, 2009; Chandler et al., 2005; Girin et al., 2010). Some of these genes such as *IND* and *ALC* interact with various hormonal pathways involved in auxin, gibberellins and ABA biosynthesis and regulate pod shattering (Sorefan et al., 2009; Arnaud et al., 2010). To our best knowledge, loci associated for natural variation for pod shatter resistance in *B. carinata* have not been identified yet.

This study aims to (i) characterize genetic variation for pod shatter resistance in *B. carinata* accessions, (ii) identify the QTL associated with pod strength in an F_2_ population and a set of 83 accessions, and (iii) determine the physical location of associated QTL on the *B. nigra* (BB genome), *B. juncea* (AB genome), *B. oleracea* (CC genome), and *B. napus* (AC genome) genomes to identify candidate genes underlying shattering resistance in *B. carinata*.

## Materials and Methods

### Plant Materials

A diversity panel of 200 accessions of *Brassica* and related species including *B. carinata* (83), *B. rapa* (90), one accession each of *B. barrelieri*, *B. deflexa*, *B. juncea*, *B. maurorum*, *B. oxyrrhina*, *B. ruvo*, *B. tournefortii*, *E. sativa*, *M. longipetala*, *S. alba*, and *S. erysimoides*, two accessions each of *A. thaliana*, *B. nigra*, *B. napus*, and *S. arvensis* and eight accessions of *B. oleracea* were obtained from the Australian Grains Genebank, Horsham (Raman R. et al., 2014). In addition, the F_2_ population comprising 300 individuals was developed from a single F_1_ cross between BC73526 (shatter resistant with high RE) and BC73524 (shattering prone with low RE) to identify the QTL associated with pod shatter resistance. Both parental lines were selected on the basis of their contrasting rupture energy values among 83 accessions of *B. carinata*. Each F_2_ line was selfed to generate F_2:3_ population to confirm phenotypes.

### Evaluation for Pod Shatter Resistance

The diversity panel comprising 200 accessions was grown in white plastic pots (10 inch diameter, Garden Plastic city, Australia) in 2012 and 2013 at the Wagga Wagga Agricultural Institute, New South Wales, Australia. Both trials consisted of a 4 range by 100 row array with two replications. Five plants were grown per pot. Passport data on days to first flowering (first open flower on at least two plants in a pot) were recorded. At maturity, five pods from each plant were collected to evaluate for shatter resistance using the pendulum test as described previously (Raman H. et al., 2014). Pod length from each test sample was measured with a scale excluding the length of ‘beak’ to adjust the position of the pod when pendulum strikes. In the present study, we only focused on 83 *B. carinata* accessions for pod shatter resistance (**Table 1**).

**Table 1:** Natural variation for pod shatter resistance in *Brassica carinata* accessions grown under birdcage conditions in 2012 and 2013.

Species	AGG accession ID	Square root predicted means for RE (mJ)	Square root SE	Backtransformed predicted means for RE (mJ)	Square root predicted means for RE (mJ)	Square root SE	Backtransformed predicted means for RE (mJ)
			
		2012			2013		
*B. carinata*	ATC90258	1.99	0.18	3.94	1.59	0.19	2.53
*B. carinata*	ATC90259	3.29	0.15	10.82	2.50	0.18	6.27
*B. carinata*	ATC90260	2.50	0.22	6.26	2.42	0.25	5.84
*B. carinata*	ATC90261	3.10	0.19	9.61	2.73	0.18	7.45
*B. carinata*	ATC90262	2.63	0.16	6.94	2.83	0.18	8.02
*B. carinata*	ATC90263	1.80	0.34	3.22	1.73	0.18	2.99
*B. carinata*	ATC90264	2.94	0.20	8.63	2.38	0.23	5.67
*B. carinata*	ATC90265	2.96	0.15	8.73	2.45	0.18	6.01
*B. carinata*	ATC90266	2.59	0.15	6.73	2.43	0.18	5.91
*B. carinata*	ATC93184-1	2.68	0.15	7.18	2.33	0.18	5.44
*B. carinata*	ATC93879	3.02	0.16	9.12	1.93	0.18	3.74
*B. carinata*	ATC93881	2.29	0.16	5.22	2.72	0.18	7.37
*B. carinata*	BC73524	3.16	0.20	9.96	2.18	0.18	4.77
*B. carinata*	ATC93884	2.39	0.20	5.73	2.11	0.18	4.43
*B. carinata*	ATC93885	2.24	0.16	5.04	2.02	0.18	4.08
*B. carinata*	ATC93886	2.16	0.16	4.68	2.76	0.18	7.62
*B. carinata*	ATC93887	2.54	0.16	6.47	2.46	0.18	6.04
*B. carinata*	ATC93888	2.08	0.26	4.32	1.95	0.18	3.81
*B. carinata*	ATC93889	1.53	0.15	2.33	2.68	0.18	7.21
*B. carinata*	ATC93890	3.16	0.22	9.98	2.45	0.19	6.00
*B. carinata*	ATC93892	2.28	0.15	5.21	2.29	0.18	5.27
*B. carinata*	ATC93895	2.88	0.22	8.28	2.14	0.19	4.58
*B. carinata*	ATC93896	2.19	0.25	4.78	1.88	0.18	3.55
*B. carinata*	ATC93897	2.26	0.34	5.12	-	-	-
*B. carinata*	ATC93898	2.63	0.23	6.91	2.69	0.19	7.24
*B. carinata*	ATC93899	2.33	0.34	5.44	2.27	0.19	5.14
*B. carinata*	ATC93954	3.08	0.24	9.50	2.94	0.25	8.65
*B. carinata*	ATC93969	2.70	0.17	7.31	2.76	0.19	7.59
*B. carinata*	ATC93971	3.46	0.15	11.96	3.83	0.18	14.67
*B. carinata*	ATC93972	3.29	0.21	10.79	3.05	0.19	9.28
*B. carinata*	ATC93973	2.06	0.22	4.24	2.09	0.21	4.35
*B. carinata*	ATC93974	2.51	0.17	6.28	2.52	0.20	6.35
*B. carinata*	ATC93975	3.52	0.18	12.37	3.16	0.18	10.01
*B. carinata*	ATC93976	2.69	0.22	7.24	2.68	0.19	7.19
*B. carinata*	ATC93977	2.97	0.18	8.80	2.64	0.22	6.95
*B. carinata*	ATC93978	3.27	0.27	10.69	3.26	0.20	10.61
*B. carinata*	ATC94009	2.39	0.16	5.73	2.32	0.20	5.37
*B. carinata*	ATC94010	2.39	0.15	5.72	2.82	0.18	7.96
*B. carinata*	ATC94011	2.56	0.15	6.53	1.96	0.18	3.84
*B. carinata*	ATC94023	3.15	0.22	9.92	3.09	0.20	9.55
*B. carinata*	ATC94024	2.20	0.34	4.85	2.49	0.25	6.19
*B. carinata*	ATC94025	-	-	-	3.04	0.28	9.24
*B. carinata*	ATC94035	3.49	0.20	12.17	3.19	0.22	10.15
*B. carinata*	ATC94037	2.91	0.22	8.48	2.72	0.25	7.43
*B. carinata*	ATC94039	3.23	0.22	10.40	2.63	0.23	6.92
*B. carinata*	ATC94041	2.84	0.27	8.09	3.45	0.20	11.90
*B. carinata*	ATC94042	3.28	0.26	10.76	1.91	0.18	3.65
*B. carinata*	ATC94043	3.27	0.17	10.70	2.90	0.18	8.41
*B. carinata*	ATC94044	2.65	0.15	7.00	2.27	0.18	5.15
*B. carinata*	ATC94045	3.29	0.18	10.80	3.02	0.18	9.12
*B. carinata*	ATC94046	3.10	0.16	9.63	3.69	0.18	13.63
*B. carinata*	ATC94047	3.54	0.15	12.50	3.20	0.19	10.26
*B. carinata*	ATC94048	2.66	0.16	7.09	2.64	0.18	6.97
*B. carinata*	ATC94049	2.98	0.34	8.91	2.53	0.18	6.38
*B. carinata*	ATC94050	2.14	0.34	4.58	2.41	0.20	5.80
*B. carinata*	ATC94109	2.96	0.19	8.79	3.12	0.21	9.72
*B. carinata*	ATC94111	3.06	0.20	9.37	2.01	0.18	4.03
*B. carinata*	ATC94113	2.77	0.20	7.70	3.02	0.25	9.14
*B. carinata*	ATC94114	3.32	0.21	11.03	2.41	0.28	5.83
*B. carinata*	ATC94116	2.41	0.27	5.83	2.56	0.40	6.56
*B. carinata*	ATC94117	2.26	0.22	5.12	2.30	0.18	5.31
*B. carinata*	ATC94119	2.85	0.17	8.11	2.89	0.19	8.38
*B. carinata*	ATC94120	1.76	0.18	3.08	1.96	0.19	3.84
*B. carinata*	ATC94125	2.28	0.16	5.21	2.58	0.18	6.66
*B. carinata*	ATC94126	4.01	0.17	16.11	4.44	0.20	19.75
*B. carinata*	ATC94134	2.67	0.15	7.15	2.15	0.18	4.64
*B. carinata*	ATC94135	2.41	0.15	5.82	1.88	0.19	3.55
*B. carinata*	ATC94137	2.46	0.17	6.05	1.80	0.18	3.24
*B. carinata*	ATC94138	2.06	0.21	4.26	2.51	0.18	6.30
*B. carinata*	ATC94139	2.90	0.16	8.43	1.89	0.18	3.56
*B. carinata*	ATC94192	1.91	0.24	3.64	2.05	0.19	4.22
*B. carinata*	ATC94409	2.08	0.38	4.33	2.70	0.22	7.30
*B. carinata*	ATC94411	2.66	0.34	7.08	2.34	0.21	5.47
*B. carinata*	ATC94416	2.82	0.26	7.93	2.45	0.22	6.01
*B. carinata*	ATC94427	2.15	0.34	4.60	-	-	-
*B. carinata*	ATC94429	3.24	0.22	10.51	2.68	0.18	7.17
*B. carinata*	ATC94455	2.27	0.18	5.15	2.22	0.18	4.91
*B. carinata*	ATC94457	4.50	0.17	20.26	4.56	0.19	20.82
*B. carinata*	ATC94458	4.22	0.15	17.83	4.55	0.18	20.74
*B. carinata*	ATC94461	2.46	0.18	6.04	2.45	0.20	5.99
*B. carinata*	ATC94463	2.39	0.22	5.73	1.89	0.23	3.58
*B. carinata*	ATC95065	2.60	0.19	6.74	2.59	0.20	6.70
*B. carinata*	ATC95199	2.17	0.15	4.72	2.16	0.18	4.67
*B. napus*	BLN2762	1.68	0.16	2.82	1.47	0.18	2.16


**Brassica carinata* lines were accessed from the Australian Grains Genebank (AGG), Horsham. *B. napus* accession, BLN2762 was accessed from the Australian National *Brassica* Germplasm Improvement Program, Wagga Wagga. Pod shatter resistance was tested with pendulum test and expressed as rupture energy (RE) in Millijoule (mJ). RE values were initially square rooted and then back transformed.*

*SE and -, represent to standard error (SE) and missing data, respectively.*

The two parental lines and their F_2_ population of 300 plants were grown in 2015 in white plastic pots (10 inch diameter, Garden Plastic city, Australia) under birdcage conditions at the Wagga Wagga Agricultural Institute, New South Wales, Australia. Plants were watered daily, fertilized weekly using in-line liquid fertilizers, and protected from aphids. A total of 71 F_2_ plants showed abnormal phenotypes with flower sterility; these individuals were discarded from genetic analysis. Five pods from 229 F_2_ plants (normal phenotype) were collected in the 50 mL tubes containing a silica sachet for further testing of pod rupture energy. Days to flowering was recorded daily for each F_2_ plant. All 229 F_2_ plants were enclosed with pollination bags to get pure F_3_ progenies, while leaving the primary stem out for the natural pod development for shatter testing. Ten F_3_ plants from 229 F_2_ families were grown in 2016 in a 20 row × 12 column array design including nine controls and two parents at Wagga Wagga. Five pods were collected per F_3_ plant. For validation, 58 F_2:3_ families (29 high RE and 29 low RE) and parents were tested with pendulum test as described earlier (Raman H. et al., 2014).

### Microscopic Analysis of Pod Anatomy

Anatomical features of pod DZ were observed in 30 random F_2_ plants and five F_2:3_ progenies from 20 F_2_ plants selected on the basis of their rupture energy (10 each with low RE and high RE). Pods were collected at 35–40 days after anthesis. Hand sections were prepared from one cm from the pedicel end of the pod. Fresh sections were observed for autofluorescence using a fluorescence microscope at the Charles Sturt University, Wagga Wagga. Photographs were taken using a Zeiss Axiphot microscope fitted with a Sony Cyber-shot digital camera.

### Statistical Analyses of Phenotypic Data

The rupture energy data of an F_2_ population and of a set of 83 diversity lines were square-root transformed to normalize and further analyzed using ASREML in R. Genotype was considered as a fixed effect and environment as random effects. The estimated means for each genotype were used for further genome-wide association analysis. The correlation between rupture energy in 2012 and 2013 was calculated using Pearson’s correlation coefficient. RE of five pods of each F_2_ plant was averaged and used for QTL analysis.

### DNA Isolation and Genotyping

Young leaf tissue of the field grown plants was collected for DNA isolation. Tissue were ground in liquid nitrogen and extracted using a method described in Raman et al. (2005). The diversity panel of 83 *B. carinata* accessions and the F_2_ population comprising 300 lines were genotyped with the genotyping-by-sequencing based DArTseq marker approach (Raman H. et al., 2014) at the DArT P/L, University of Canberra, Australia.

### Genetic Relatedness and Population Structure

In order to determine molecular diversity in *B. carinata*, we genotyped 83 accessions with high-quality DArTseq markers having call rate of ≥90%, ≤5% of missing data and minor allele frequency (MAF) of >0.05. Hierarchical cluster analysis based on Euclidian distance was conducted using the software, PRIMER 6 (Clarke and Gorley, 2006). Principal coordinate analysis was performed to understand the global diversity among accessions. Bayesian clustering was performed to infer the number of sub-populations among 83 accessions using the software package STRUCTURE v 2.3.4 (Pritchard et al., 2000). The program was run using admixture model with correlated allele frequencies. The presumed sub-population number (*k*) was set from 1 to 5. Ten runs for each *k* were performed with 20,000 burn in period and 50,000 Markov Chain Monte Carlo iterations per run, with no prior information on the origin of individuals. The best *k* value was determined by using the (i) logarithm likelihood for each *k* [L(*k*)], (ii) an *ad hoc* quantity (Δ*k*) according to Rosenberg et al. (2001) and Δ*k* method described by Evanno et al. (2005), respectively. Genotypes were classified into subpopulations based on their membership coefficients estimated in STRUCTURE.

### Map Construction and QTL Identification for Pod Shatter Resistance

The linkage map of F_2_ population was constructed using DArT P/L’s OCD MAPPING program (Petroli et al., 2012). Markers were clustered into linkage groups according to the method described by Wu et al. (2008). Markers with identical genotypes are placed in redundant bins, and the resulting markers/bins within each linkage group were ordered using the traveling salesman path solver program Concorde (Applegate et al., 2006). The linkage map was constructed for each parent by combining the relevant *in silico* DArT and SNP markers. A linkage map was chosen to be seed map and then a consensus map was constructed using the markers in common for the complete F_2_ population.

Two QTL mapping strategies implemented in software packages, GAPIT in the R (Lipka et al., 2012) and SVS (Golden Helix, Bozeman, MT, United States) were used to identify loci associated with pod shatter tolerance. For GAPIT analysis, we did not correct population structure using principal components in the F_2_ mapping population. Linear marker regression analysis was performed to determine trait-marker associations in the SVS package. The same approach was also followed to reveal the genome-wide association between DArTseq markers and rupture energy among 83 accessions. For GWAS, we selected a set of 54,034 high quality markers which were genotyped across all accessions. To control spurious trait-marker associations, the first 10 eigenvectors (principal components) were calculated in the SVS package. Cryptic relatedness due to ancestry by descent was controlled with the Identity-by-Decent matrix (K matrix). The Mixed Linear Model (Price, 2006; Yu et al., 2006) adjusted with K-matrix and population structure matrix with PC1 – PC10 was used to test the trait-marker associations in the SVS package. The *p*-values were adjusted to control the false discovery rate (FDR) of 5%. The significance threshold was determined by applying Bonferroni correction [*p* = 0.05/6464 (total of markers mapped): 7.73515E_-06_]. Trait-markers with significance ≤ log(_10_)*p* of 5.11153 were ‘declared’ as true associations for pod shatter resistance in an F_2_ population. Manhattan plots were generated in the SVS package.

### Alignment of Markers with the *Brassica* Reference Genomes

The physical map positions of significant markers associated with pod shatter resistance were determined using the reference *B. nigra, B. oleracea*, *B. juncea*, and *B. napus* genomes by BlastN (Altschul et al., 1990) searches, as detailed in Raman H. et al. (2014). The physical positions of pod shatter resistance genes in *A. thaliana* (accessed from TAIR^1^) were also determined by searching sequence identities with the reference genomes. The top blast significant hits (≥E^-10^) were considered to infer the putative physical positions of markers/candidate genes on the reference genomes, while blast hits to multiple loci with the same top E value were considered to be unmapped onto the reference genome.

## Results

### Phenotypic Variation for Pod Shatter Resistance in *B. carinata* Accessions

**FIGURE 1:**
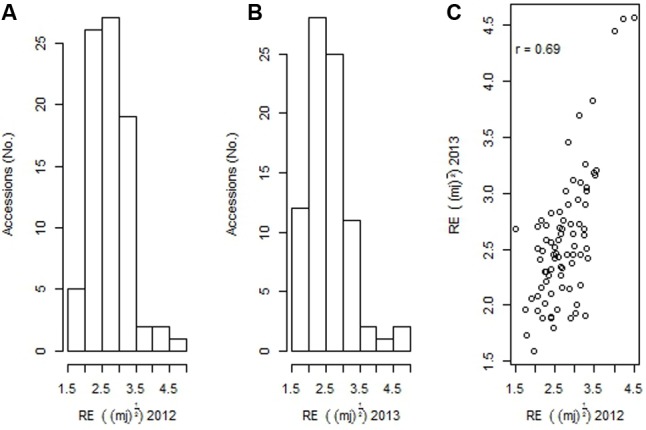
**(A)** Natural variation for pod shatter resistance in 83 accessions of *Brassica carinata* evaluated under 2012 and **(B)** 2013 environments, and **(C)** correlation of rupture energy (RE) scores of 83 accessions evaluated under 2012 and 2013 environments. Pod shatter resistance was measured with pendulum test as RE. RE presented for different accessions are square root transformed.

There were significant differences (*p* < 0.001) within the 83 *B. carinata* accessions tested with respect to pod rupture energy that ranged from 1.52 to 4.5 mJ in 2012, and 1.6 and 4.6 mJ in 2013 (**Figures 1A,B**). A positive strong correlation (*r* = 0.69) among accessions evaluated across both the 2012 and 2013 growing environments was observed, indicating that RE is genetically controlled (**Figure 1C**). Three *B. carinata* accessions, ATC94126, ATC94457, and ATC94458 had 9.14 to 9.63 times higher RE compared to the *B. napus* control genotype, BLN2762 (**Table 1**).

### Genetic Diversity and Population Structure

**FIGURE 2:**
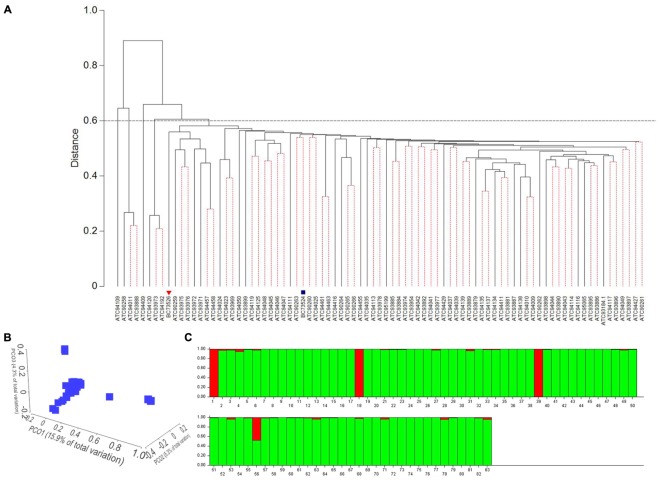
Molecular diversity among *B. carinata* accessions revealed by 54,034 DArTseq markers. **(A)** Dendrogram of 83 *B. carinata* accessions based on Euclidean distance. A total of 1,000 bootstraps were performed. Clusters with dotted lines were non-significant at the 5% level of significance. Parental lines, BC73526 and BC732524 are markers with inverted triangle (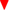) and square (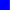), respectively. **(B)** A 3D plots of the first three principal coordinates (PCO) of (PCO1, PCO2, and PCO3) showing distribution of the *B. carinata* accessions. The proportion of variation by these axes is given in *parentheses.* **(C)** Population structure of *B carinata* accessions by STURUCTURE. Each accession is represented by a *vertical bar* (labeled as 1 to 83, representing different accessions; detailed in **Table 1**). Red and light green color bars represent to two subpopulations I and II, respectively. Number of subpopulations were determined on Δ*k* [the rate of change of LnP(D)] as shown in **Supplementary Figure S1**.

A set of 54,037 high quality DArTseq markers with call rates of >90% and a reproducibility of >95% were selected for genetic diversity and population structure analyses to determine whether shatter resistant sources are genetically diverse (**Table 1**). Hierarchical cluster analysis based on the Euclidean distance revealed five distinct groups at 60% similarity (**Figure 2A**). The cluster I was the largest with 75 accessions, followed by three accessions in cluster II (ATC94120, ATC93973, and ATC94192) and cluster IV (ATC90258, ATC94011, and ATC93888). Both cluster III (ATC94409), and cluster V (ATC94109) contained only one accession (**Figure 2A**). The overall genetic diversity among accessions was assessed with PCO analysis (**Figure 2B**), which revealed similar clustering. There were four clear groups with the majority of the accessions in cluster I. The first three coordinates (PC1 = 15.9%, PC2 = 5.3%, and PC3 = 4.3%) accounted a total of 25.39% of the genetic variation (**Figure 2B**), suggesting a weak population structure. The Bayesian – based clustering analysis using the maximum likelihood distribution LnP(D) of 83 accessions identified two sub-populations as shown in **Figure 2C**. The Wilcoxon test also revealed the presence of two subpopulations. Seventy nine accessions were in sub-population I and four accessions were in sub-population II. The STRUCTURE analysis supported the results of cluster analysis; all 83 accessions were grouped in two clusters at 90% similarity (**Figure 2A**).

### Genetic Variation and Inheritance for Pod Shatter Resistance

**FIGURE 3:**
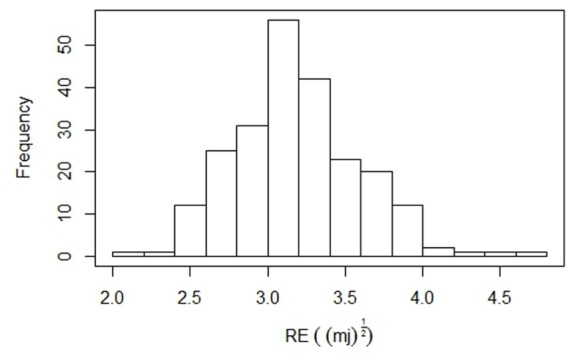
Frequency distribution of pod shattering scores (rupture energy) in the F_2_ segregation population, containing 229 individuals, derived from BC73526/BC73524. The average RE scores of the parental lines, BC73526 and BC73524 are indicated by solid arrows.

Based on the pod shatter resistance (RE) scores, two single plant selections were made from accessions BC73526 (high RE) and BC73524 (low RE) to generate an F_2_ population, representing cluster I (**Figure 2A**). Both parental lines of the F_2_ mapping population from the cross, BC73524/BC73526 differed significantly from each other with respect to pod shatter resistance; the shatter prone, maternal parent (BC73524) had the lower RE of 2.2 mJ^(1/2)^ (4.8 mJ) and the resistant, paternal parent (BC73526) had the higher RE of 4.4 mJ^(1/2)^ (19.8 mJ; **Table 1**). The F_2_ population showed a continuous distribution of RE scores, ranging from 2.2 to 4.7 mJ^(1/2)^ with the mean score of 2.71 mJ^(1/2)^ (**Figure 3**). This was typical for quantitative traits such as pod shattering resistance. In order to validate these F_2_ RE scores, we evaluated the F_2:3_ progenies (Supplementary Table S1). Our results showed that there was a strong positive correlation (*r* = 0.83) between RE scores of F_2_ plants and their F_2_:_3_ progenies, suggesting that phenotypic scores in F_2_ were accurate.

### Multiple Genes Control Pod Shatter Resistance in *B. carinata*

A total of 6,464 markers that showed polymorphism between the parents, and segregated in the complete set of F_2_ population (300 lines) were selected for the genetic linkage map construction (Supplementary Table S1). All of the mapped markers were assigned to the 17 linkage groups, equivalent of haploid genome of *B. carinata*. Of them, 4,981 marker loci were located on the 8 linkage groups of B_c_ subgenome and 1,483 loci were on the 9 linkage groups of C_c_ subgenome, covering a total genetic distance of 1622.82 cM (**Table 2**). The marker density ranged from 1.07 (C4) to 7.35 (B01) with an average density of 3.98 cM. Chromosome C5 had the least number of markers (78) as compared to B4 (881). This genetic linkage map was further used for the QTL identification.

**Table 2:** Summary of segregating markers and their coverage on the linkage genetic map of the F_2_ population derived from the BC73524/BC73526 of *B. carinata*.

Chromosome	Mapped	Map length	Average
	markers	(cM)	marker
			density/cM
B1	835	113.61	7.35
B2	634	128.87	4.92
B3	591	117.05	5.05
B4	881	148.70	5.92
B5	667	130.15	5.12
B6	386	90.28	4.28
B7	445	93.27	4.77
B8	542	141.86	3.82
Subtotal of Bc subgenome	4981	963.80	5.17
C1	120	68.19	1.76
C2	113	36.09	3.13
C3	348	109.79	3.17
C4	94	88.12	1.07
C5	78	26.41	2.95
C6	212	71.98	2.95
C7	130	65.11	2.00
C8	120	82.94	1.45
C9	268	110.40	2.43
Subtotal of the Cc subgenome	1483	659.04	2.25
Total of the B_c_C_c_ genome	6464	1622.842	
Mean	380.23	95.46	3.98


*LG were assigned to eight chromosomes of the B_*C*_ subgenome and nine chromosomes of the C_*C*_ subgenome of *B. carinata* on the basis of their physical map locations on the reference genomes of *B. nigra, B. juncea* cv. Tumida (for the B subgenome), *B. oleracea* (T1000) and *B. napus* cv. Darmor (for the C subgenome).*

**FIGURE 4:**
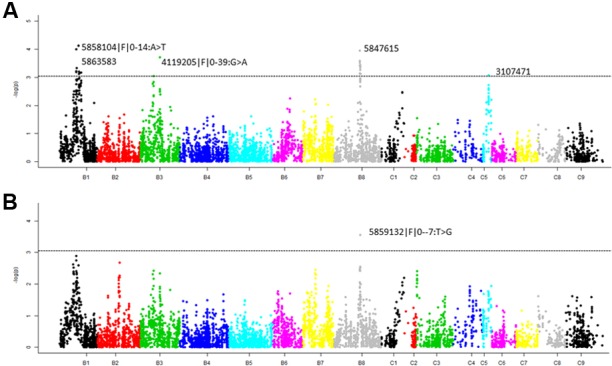
Manhattan plots showing **(A)** marker- pod shatter resistance associations and **(B)** marker-pod length associations, in the F_2_ population from BC73524/BC73526 using GAPIT analysis. Highly significant markers are also shown; marker depicted with >denotes DArTseq SNP markers and without >symbol denotes *in silico* DArT. The suggestive threshold LOD value (3.0) for trait-marker association is shown as dashed line.

We identified five significant QTL (LOD = 3) associated with pod shatter resistance, *Qpsr.wwai-B1a, Qpsr.wwai-B1b*, *Qpsr.wwai-B3*, *Qpsr.wwai-B8*, and *Qpsr.wwai-C5* in the BC73524/BC73526 population (**Table 3**, **Figure 4A** and Supplementary Table S1). Two QTL; *Qpsr.wwai-B1a* tagged with the *in silico* DArT marker 5863583, and *Qpsr.wwai-B1b* tagged with DArTseq-SNP marker 5858104|F| 0-14:A > T, were located 7.4 cM apart on chromosome B1. Other three QTL, *Qpsr.wwai-B3*, *Qpsr.wwai-B8*, and *Qpsr.wwai-C5* were identified on chromosomes B3, B8, and C5, respectively (**Table 3**). Of these, *Qpsr.wwai-B1b* accounted for the maximum (5.27%) of the phenotypic variation and the *Qpsr.wwai-C5* accounted for the least (3.71%). All five QTL explained a total of 23.73% of the phenotypic variation for RE. The shatter resistant parent, BC73526 contributed the favorable allele as envisaged by pod strength, and thus reduced pod shattering in progenies. DArTseq markers were assigned the physical positions on *B. carinata* genome, by comparing their sequence identities with the reference genomes of *B. nigra*, *B. juncea, B. oleracea*, and *B. napus*. Our results showed that the *Qpsr.wwai-B1a, qPSR.wwai-B1b*, *Qpsr.wwai-B3*, *Qpsr.wwai-B8*, and *Qpsr.wwai-C5* were located to the pseudomolecules of B1, B3, B8, and C5, respectively (**Supplementary Figure S2** and Table S2).

**Table 3:** Quantitative trait loci (QTL) associated with pod shatter resistance in the F_2_ population from the BC73526/BC73524.

QTL	Highly significant marker	Chromosomal location	Chromosomal position	LOD score	*R*^2^ (%)	*Brassica* reference genome	Physical map position (bp)	Nearest candidate gene for pod shatter resistance from significant SNP association	Physical distance between SNP and candidate gene (kb)
*Qpsr.wwai-B1a*	5863583	B1	49.8	9.86E-05	5.09	*B. nigra*/CM004491.1“_B1	19,563,260	*FUL*	63.12
*Qpsr.wwai-B1b*	#5858104|F| 0-14:A > T	B1	57.2	7.45E-05	5.27	CM007195.1“_B1	Unknown	*FUL*	8263.23
*Qpsr.wwai-B3*	4119205|F|0-39:G > A	B3	58.5	0.0002	4.65	*B. nigra* CM0044931.1“_B3	32,518,934	*IND*	2493.1
*Qpsr.wwai-B8*	5847615	B8	77.0	0.0001	5.01	*B. nigra*/CM004498.1“_B8	31,742,473	*RPL*	1131.55
*Qpsr.wwai-C5*	3107471	C5	16.2	0.000832	3.71	*B. oleracea*“_/C_n_5	11,396,951	*FUL*	454.62


*Reference genomes of *B. nigra*, *B. juncea* cv. tumida, version 1.0, *B. oleracea* (T1000) and *B. napus* version 4.1, were used for sequence alignments against *B. carinata* sequences. Details of alignments with the pseudomolecules of *B. juncea* and *B. napus* are given in the Supplementary Table S2. #Appropriate physical location based on the bin markers.*

To establish whether pod length relates to pod shattering, we mapped QTL associated with pod length in the F_2_ population. Our results showed that one significant marker, 5859132|F| 0–7:T > G at QTL (LOD = 3.55) was associated with pod length in the F_2_ population (**Figures 3**, **4B**, and Supplementary Tables S2, S3). This QTL was identified on chromosome B8 and mapped 1 cM apart from the pod shatter resistance QTL, *Qpsr.wwai-B8*. Two other markers, 5832583 and 5863583 on chromosome B1 also showed association with pod length (LOD scores of 2.7 to 2.9, Supplementary Table S3).

GWAS analysis using 54,034 markers based polymorphisms was performed to verify the alleles for pod shatter resistance in diverse *B. carinata* accessions Although, we used a small number of accessions for this analysis, we found 19 statistically significant SNP associations between markers and pod shatter resistance (RE scores) based on the Bonferroni corrected threshold –log 10(*p*) = 9.25292E^-07^ (Supplementary Table S3). By controlling type 1 error using kinship coefficients (IBS) and first 10 principal components at least 16 consistent significant associations were identified across both 2012 and 2013 trials with LOD score of ≤5.35 (Supplementary Tables S3, S4).

### Physical Mapping of Significant QTL and Alignment with *Brassica* Reference Genomes

Of 6464 DArTseq markers mapped, the chromosomal positions of 5,080 markers could be linked with the pseudomolecule positions to the published genome sequences of *B. oleracea*, *B. napus, B. juncea*, and *B. nigra* (Supplementary Table S1). We also anchored several scaffolds which have been unmapped yet to the pseudomolecules of *B. juncea* genome assembly in an F_2_ population. Furthermore, marker sequences targeting QTL were aligned with the sequenced reference B, C and AC genomes and physical intervals harboring candidate genes for pod shatter resistance. Of the seven pod shatter resistance genes of *A. thaliana* searched, *FUL –* a MADS box gene negatively regulated by *APETALA1* (TAIR ID: AT5G60910.1), was located 63.1 kb away from the significant SNP marker, 5863583 on chromosome 1B (**Table 3**). Other candidate genes were located 0.4 to 8.3-Mbp apart from corresponding QTL regions in the F_2_ population (Supplementary Tables S4, S5). We identified 40 GWAS SNP associations (LOD ≤ 3) in the proximity (5.1 kb to 16 Mbp) of genes controlling pod shattering in *A. thaliana*. Of them, three orthologs of *FUL* were located on chromosome B1 (5.1 kb), B6 (97.4 kb) and on the LFLV01001230.1scaffold_28.1 of the reference genome of *B. nigra* (34.69 kb), while two orthologs of *IND* were located on chromosome B1 (53.69 kb) and B2 (96.77 kb). One ortholog of *SHP2* was also identified within 77 kb region of chromosome B5 corresponding to SNP association with 100067358|F| 0-31:T > C marker (Supplementary Table S5).

### Pod Shatter Resistance Is Related with Pod Dehiscence Zone Differentiation in *B. carinata*

**FIGURE 5:**
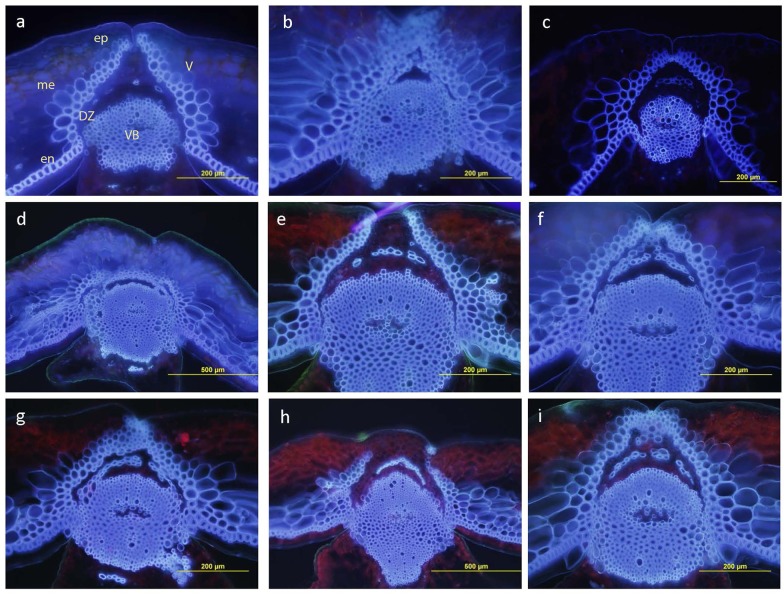
Anatomical features of dehiscence zone also called abscission layer in parents and F_2_ plants of the BC73524/BC73526. **(a)** BC73524, **(b)** BC73526, **(c)** *B. napus* advanced breeding line, BLN2762 and **(d–i)** F_2_ plants with varying level of dehiscence zone development. Transverse pod section of BC73524 showing well-developed DZ whereas BC73526 showing almost no DZ differentiation. V: valve, VB: vascular bundle of ruplum, en: endocarp, ep: epicarp, me: mesocarp.

Pod structure was observed (40 days after anthesis) under a fluorescence microscope to determine any link between the pod DZ differentiations and shatter resistance in *B. carinata*. The anatomical feature of parents displayed a distinctive difference in the valve margin formation (**Figure 5**). The shatter prone parent, BC73524 had the well-developed DZ comprising thin walled parenchymatous cells (**Figure 5a**) compared to the shatter resistant parent, BC73526 (**Figure 5b**). Thirty randomly selected F_2_ plants exhibited a varied level of DZ development pattern (**Figures 5d–i**). For example, there was either clear DZ along the whole valve margin (**Figure 5e**), similar to shatter prone parent (**Figure 5a**); loss of DZ proximal to the main vascular bundle (mv) as well as near the outer part of the replum (**Figure 5d**), similar to the shatter tolerant parent (**Figure 5c**); and DZ proximal to the main vascular bundle (mv) but did not extend near the outer part of the replum (**Figure 5h**). In *B. napus*, a well-developed DZ was clearly evident (**Figure 5c**) similar to the shatter prone *B. carinata* parent, BC73524.

## Discussion

Considering the commercial value of oilseed *Brassica* crops (*B. napus*, *B. rapa*, and *B. juncea*) worldwide, genetic improvement for pod shatter resistance is of paramount importance to reduce unwanted losses. Despite of limited genetic diversity in *B. carinata* germplasm (Jiang et al., 2007; Guo et al., 2012), several accessions were found to be useful in uncovering genetic variation for resistance to pod shatter (**Table 1**). For example, we found three accessions which had more than nine times higher pod RE as compared to the *B. napus* control genotype, BLN2762. Genetic variation in these accessions could be harnessed for further genetic improvement of *B. carinata* as well as other *Brassica* species.

We determined the pod strength (RE) in *B. carinata* with pendulum test, as a proxy for shatter resistance. This method was found to be reliable and repeatable in determining the extent of pod-shatter resistance and mapping QTL in *B. carinata* (this study, **Table 3** and Supplementary Table S4). Similar findings were made in previous studies on genetic variation for pod shatter resistance in *B. rapa* and *B. napus* (Kadkol et al., 1985, 1986b; Prakash and Chopra, 1988; Mongkolporn et al., 2003; Wang et al., 2007; Raman H. et al., 2014; Zhang et al., 2016). We revealed that pod shatter resistance is due to multiple genes in the F_2_ population of *B. carinata* derived from the BC73524/BC73526. Multigenic inheritance for pod shatter resistance in *B. carinata* (this study) is consistent with previous findings in *B. rapa* and *B. napus* (Mongkolporn et al., 2003; Wen et al., 2013; Raman H. et al., 2014; Liu et al., 2016).

In this study, a linkage map of a F_2_ population was constructed utilizing 6,464 DArTseq markers and subsequently used for QTL analysis. The marker density of this linkage map (**Table 2**) was comparable with the linkage map of a DH population of *B. carinata* derived from YW (Zou et al., 2014). The majority of DArTseq markers were linked with the physical positions on the reference genomes of *B. nigra/B. juncea* and *B. oleracea*/*B. napus*. In addition, several scaffolds which were unassembled in the reference *B. juncea* sequence (Yang et al., 2016) could be mapped to the linkage map of *B. carinata* population (Supplementary Table S1). Our results suggested that the reference genomes are useful in anchoring different linkage groups to pseudomolecules and facilitating molecular marker and candidate gene discovery. One of the QTL, *Qpsr.wwai-B1a* delimited with marker 5834957 was mapped to the B1 pseudomolecule of *B. nigra* within 63.12 kb of Arabidopsis *FUL* ortholog (Supplementary Table S4). Ostergaard et al. (2006) showed that ectopic expression of the Arabidopsis *FUL* gene in *B. juncea* is sufficient to produce pod shatter resistance, via negative regulation of the valve-margin identity genes (Ferrandiz et al., 2000). However, the transgenic *B. juncea* fruit produced were too tightly closed. Similar observations were made in this study, the shatter resistant accession BC73526 did not dehisce under natural field conditions and there was no clear separation between valve margin and replum (**Figure 5**). A close link between pod shatter resistance and DZ differentiation was observed, the shatter prone and shatter tolerant accessions could be differentiated based on pod anatomy. The shatter prone accession (BC73524) had a well-developed DZ as compared with shatter resistant (BC73526). Similar observations have been made in *A. thaliana*, *B. rapa*, *B. napus*, and *B. carinata* (Kadkol et al., 1986a; Jenkins et al., 1999; Ferrandiz et al., 2000; Sorefan et al., 2009; Raman H. et al., 2014). Several genes; *FUL*, *SHP1*, *SHP2*, *ALC*, *IND*, and *RPL* have been implicated in the development of the valve-margin separation layer, and lignification of the endocarp layer (Dinneny and Yanofsky, 2005). Girin et al. (2010) showed that homozygous *braA.ind.a* mutants showed a clear loss of valve margin formation in *B. rapa* and *B. oleracea.*

The marker 5834957 at *Qpsr.wwai-B1a* also showed the complete linkage with other loci; 5861424, 5832583, 5843024, 5854441, 5842255, 5843155, and 5849931. These markers were mapped at the 49.81 cM of the F_2_ map and showed significant sequence identities with the A09 reference genome sequence of *B. juncea* (coordinates 6,440,430 to 7,118,167 CM007193.1_chromosome_A9, coordinate 6480570bp, 1.84E-25) (Supplementary Table S1). In previous studies, a major QTL for pod shatter resistance was located on chromosomes A09/C08, in the vicinity of *SHATTERPROOF* gene in *B. napus* populations (Hu et al., 2012; Raman H. et al., 2014; Liu et al., 2016). We searched the *SHP1* and *SHP2* orthologs in the reference genome of *B. juncea*. One of the *SHP1* homologs was mapped ∼35 Mb away from the highly significant SNP marker 5834957 on pseudomolecule A09 of *B. juncea* [sequence identity = 323 bits (163), Expect = 3e-86, 211/227 (92%); coordinates 45,984,225 to 45983999 (Supplementary Table S4)]. While, one of the six *SHP2a* (JQ973082.1 *B. napus SHATTERPROOF* mRNA) homologs was located in the vicinity of the highly significant SNP marker 5834957 on chromosome B1/A09 [313 bits, score: 2e-83, Identities = 176/182 (96%)]. In addition to *SHP2* and *FUL*, other genes controlling pod shatter resistance such as *IND* were also mapped near the statistical significant marker associations (**Table 3**), suggesting the markers identified for pod shatter resistance herein are reliable.

## Conclusion

We mapped QTL controlling pod shatter resistance in *B. carinata* and identified sequence-based molecular markers. These trait-marker associations with respect to reference genomes of *B. napus*, and *B. juncea* could also pave the way for delineation of pod shatter resistance QTL involved in natural variation, map-based cloning of those QTL and unravel the molecular architecture of pod shatter resistance genes in natural germplasm of *B. carinata*. In addition, molecular markers identified herein will enable us to trace the introgression of pod shatter resistance alleles for strategic improvement of *B. carinata*, *B. napus*, and other related species. Previous studies have reported that there is limited genetic variation for pod shatter resistance in the natural *B. napus* germplasm (Bowman, 1984; Raman H. et al., 2014). Several research groups around the world are currently using *B. carinata* to expand the narrow genetic base of *B. napus* germplasm (Cowling, 2007; Dhaliwal et al., 2017). In this study, only one QTL, *Qpsr.wwai-C5* was identified on the C subgenome of *B. carinata* (chromosome C05), while other QTL were identified on the B subgenome (B1, B3, and B8). Previous studies have shown that fertile plants of *B. napus* carrying B genome introgressions can be generated (Navabi et al., 2010; Dhaliwal et al., 2017). It remains to be established whether B and C genome derived lines exhibit pod shatter resistance expression or get silenced in the resynthesized *B. napus* (Xu et al., 2009). Nevertheless, our results provide valuable information on donor sources for pod shatter resistance, genetic inheritance, genetic map location of QTL, and associated markers for marker-assisted selection. The markers identified in this study can be assayed on any sequencing platform and/or converted into simple KASP assay for high throughput analysis.

## Author Contributions

RR and HR designed the study, and prepared the manuscript. RR developed F_2_ and F_3_ populations, conducted the experiments and analyzed the data. NC designed the field trials and RR and NC analyzed the data, YQ assisted in phenotyping, performed pod anatomy, and DNA extractions. AK and JS aligned DArTseq data with the reference genomes. All authors reviewed and edited the manuscript.

## Conflict of Interest Statement

AK is the director of Diversity Arrays Technology Pty Ltd. and JS was employed by Diversity Arrays Technology Pty Ltd. All other authors declare no competing interests.

## Footnotes

1 https://www.arabidopsis.org

## Supplementary Material

**FIGURE S1 |** Analysis of the population structure of 83 *B. carinata* accessions. The likelihood values [Ln(P(D] for each successive K.

**FIGURE S2 |** Manhattan plots showing marker-pod shatter resistance associations in the F_2_ population using the SVS package. A linear markers regression analysis was performed to identify loci associated with pod rupture energy.
