# Supplementary material for: Molecular Diversity Analysis and Genetic Mapping of Pod Shatter Resistance Loci in Brassica carinata L
Source: Front Plant Sci. 2017 Nov 30;8:1765. doi: 10.3389/fpls.2017.01765 (PMC5716317; doi:10.3389/fpls.2017.01765)
Supplement: Supplementary file 1 [file Data_Sheet_1.DOCX]

Supplementary Figure 1:
