# Supplementary figures and images for: Molecular Diversity Analysis and Genetic Mapping of Pod Shatter Resistance Loci in Brassica carinata L
Source: Front Plant Sci. 2017 Nov 30;8:1765. doi: 10.3389/fpls.2017.01765 (PMC5716317; doi:10.3389/fpls.2017.01765)

## Slide 1
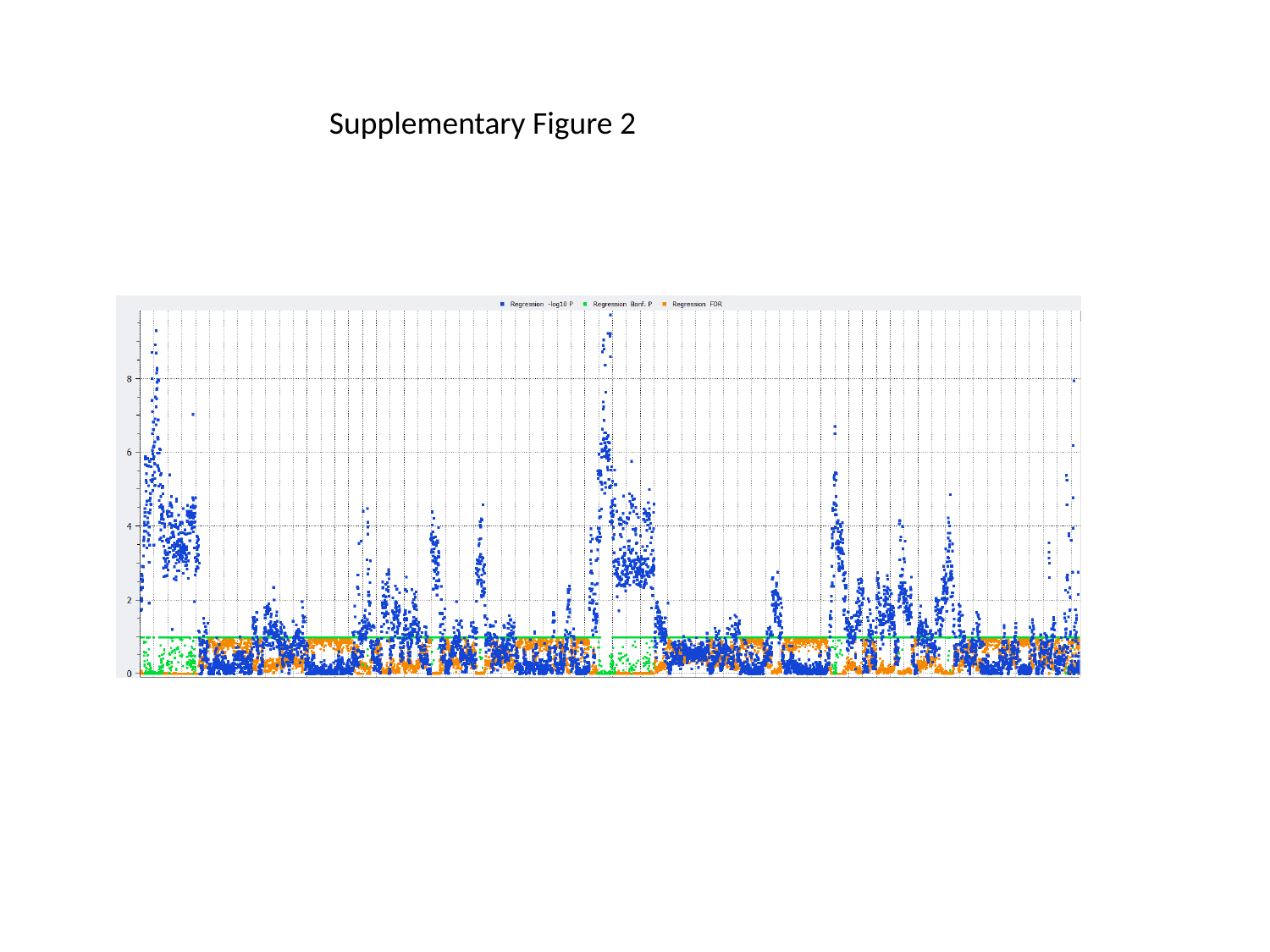

Supplementary Figure 2

Supplement: Supplementary file 2 [file Presentation_1.PPTX]
