# Supplementary material for: Molecular Diversity Analysis and Genetic Mapping of Pod Shatter Resistance Loci in Brassica carinata L
Source: Front Plant Sci. 2017 Nov 30;8:1765. doi: 10.3389/fpls.2017.01765 (PMC5716317; doi:10.3389/fpls.2017.01765)
Supplement: Supplementary file 3 [file Table_1.DOCX]

| Genotype ID | F_2_ AvrRE-2014 (mJ) | F_2:3_  AvrRE  -2015 (mJ) |  | Genotype ID | F_2_  AvrRE-2014 (mJ) | F_2:3_  AvrRE-2015 (mJ) |
| --- | --- | --- | --- | --- | --- | --- |
| 14BcF2-2pl566 | 6.4 | 6.58 |  | 14BcF2-2pl540 | 12.4 | 8.54 |
| 14BcF2-2pl577 | 6.6 | 4.78 |  | 14BcF2-2pl338 | 12.4 | 6.46 |
| 14BcF2-2pl552 | 6.7 | 7.26 |  | 14BcF2-2pl495 | 12.5 | 5.86 |
| 14BcF2-2pl599 | 7.1 | 7.16 |  | 14BcF2-2pl554 | 12.7 | 15.08 |
| 14BcF2-2pl379 | 7.5 | 6.14 |  | 14BcF2-2pl581 | 12.8 | 14.82 |
| 14BcF2-2pl399 | 7.9 | 6.54 |  | 14BcF2-2pl397 | 13.0 | 14.28 |
| 14BcF2-2pl428 | 8.4 | 7.16 |  | 14BcF2-2pl470 | 13.1 | 13.22 |
| 14BcF2-2pl422 | 8.4 | 6.92 |  | 14BcF2-2pl575 | 13.3 | 14.4 |
| 14BcF2-2pl342 | 8.5 | 7.22 |  | 14BcF2-2pl333 | 13.5 | 8.34 |
| 14BcF2-2pl302 | 8.6 | 7.5 |  | 14BcF2-2pl488 | 13.7 | 14.16 |
| 14BcF2-2pl430 | 8.7 | 6.24 |  | 14BcF2-2pl334 | 13.8 | 7.9 |
| 14BcF2-2pl306 | 9.1 | 7.02 |  | 14BcF2-2pl531 | 13.9 | 14.06 |
| 14BcF2-2pl556 | 9.4 | 11.5 |  | 14BcF2-2pl382 | 14.2 | 15.96 |
| 14BcF2-2pl425 | 9.5 | 5.7 |  | 14BcF2-2pl534 | 14.3 | 14.38 |
| 14BcF2-2pl329 | 9.7 | 6.68 |  | 14BcF2-2pl530 | 14.5 | 13.48 |
| 14BcF2-2pl451 | 9.8 | 6.06 |  | 14BcF2-2pl546 | 14.9 | 13.6 |
| 14BcF2-2pl360 | 9.8 | 7.36 |  | 14BcF2-2pl565 | 15.1 | 14.82 |
| 14BcF2-2pl504 | 10.5 | 7.32 |  | 14BcF2-2pl301 | 15.3 | 13.18 |
| 14BcF2-2pl398 | 10.6 | 7.08 |  | 14BcF2-2pl519 | 15.8 | 19.38 |
| 14BcF2-2pl352 | 10.6 | 7.46 |  | 14BcF2-2pl494 | 15.9 | 15.5 |
| 14BcF2-2pl405 | 10.7 | 7.2 |  | 14BcF2-2pl357 | 16.1 | 14.94 |
| 14BcF2-2pl404 | 10.8 | 7.6 |  | 14BcF2-2pl598 | 16.3 | 15.76 |
| 14BcF2-2pl369 | 10.9 | 7.62 |  | 14BcF2-2pl441 | 16.4 | 15.16 |
| 14BcF2-2pl474 | 11.1 | 15.48 |  | 14BcF2-2pl572 | 16.7 | 14 |
| 14BcF2-2pl567 | 11.3 | 9.28 |  | 14BcF2-2pl361 | 16.8 | 16.08 |
| 14BcF2-2pl376 | 11.8 | 14.16 |  | 14BcF2-2pl592 | 17.4 | 14.82 |
| 14BcF2-2pl476 | 11.9 | 14.04 |  | 14BcF2-2pl532 | 17.6 | 16.66 |
| 14BcF2-2pl381 | 12.1 | 7.46 |  | 14BcF2-2pl595 | 18.6 | 19.34 |
| 14BcF2-2pl589 | 12.1 | 13.36 |  | 14BcF2-2pl569 | 18.8 | 21.72 |

Supplementary Table 1: Average rupture energy (AvrRE) of selected F_2_ plants derived from the BC73526/BC73524 in 2014 and their F_2:3_ progenies in 2015
